# Cardiometabolic risk factors in MASLD patients with HCC: the other side of the coin

**DOI:** 10.3389/fendo.2024.1411706

**Published:** 2024-05-23

**Authors:** Marica Meroni, Miriam Longo, Paola Dongiovanni

**Affiliations:** Medicine and Metabolic Diseases, Fondazione IRCCS Cà Granda Ospedale Maggiore Policlinico, Milan, Italy

**Keywords:** MASLD, HCC, metabolic dysfunctions, cholesterol, carotid plaques

## Abstract

Metabolic Dysfunction-Associated Steatotic Liver Disease (MASLD) constitutes the commonest cause of chronic liver disorder worldwide, whereby affecting around one third of the global population. This clinical condition may evolve into Metabolic Dysfunction-Associated Steatohepatitis (MASH), fibrosis, cirrhosis and hepatocellular carcinoma (HCC), in a predisposed subgroup of patients. The complex pathogenesis of MASLD is severely entangled with obesity, dyslipidemia and type 2 diabetes (T2D), so far so nutritional and lifestyle recommendations may be crucial in influencing the risk of HCC and modifying its prognosis. However, the causative association between HCC onset and the presence of metabolic comorbidities is not completely clarified. Therefore, the present review aimed to summarize the main literature findings that correlate the presence of inherited or acquired hyperlipidemia and metabolic risk factors with the increased predisposition towards liver cancer in MASLD patients. Here, we gathered the evidence underlining the relationship between circulating/hepatic lipids, cardiovascular events, metabolic comorbidities and hepatocarcinogenesis. In addition, we reported previous studies supporting the impact of triglyceride and/or cholesterol accumulation in generating aberrancies in the intracellular membranes of organelles, oxidative stress, ATP depletion and hepatocyte degeneration, influencing the risk of HCC and its response to therapeutic approaches. Finally, our pursuit was to emphasize the link between HCC and the presence of cardiometabolic abnormalities in our large cohort of histologically-characterized patients affected by MASLD (n=1538), of whom 86 had MASLD-HCC by including unpublished data.

## 1 Introduction

Due to the global spreading of Metabolic Syndrome (MetS), the newly re-defined Metabolic Dysfunction-Associated Steatotic Liver Disease (MASLD) represents the greatest challenge for the global Health systems of the 21st century. This concept is even more relevant when we consider that it may constitute the etiopathological substrate for the development of advanced stages of the disease, passing towards Metabolic Dysfunction-Associated Steatohepatitis (MASH), to fibrosis, cirrhosis and hepatocellular carcinoma (HCC). As assumed by the novel nomenclature, several metabolic alterations may be causative of steatosis, thus being more inclusive of cardiometabolic risk factors as abdominal obesity, dyslipidemia, hypertension, and hyperglycemia. Indeed, the new terminology implies body mass index (BMI) >= 25 kg/m^2^ or increased waist circumference (WC >94 cm for men and 80 cm for women); raised plasma triglycerides (TGs>= 1.70 mmol/L or lipid lowering treatments); reduced HDL cholesterol (<= 1.0 mmol/L or lipid lowering agents); high blood pressure (>= 130/85 mmHg or therapy with anti-hypertensives); and enhanced fasting serum glucose (>= 100 mg/dL) or glucose intolerance (1).

A total of 10–20% of HCC cases is attributable to MASLD (2), thus projecting the proportion of the annual incidence of the liver cancer worldwide and requiring a growing attention in surveillance programs (3, 4). With the advances in antiviral therapies, MASLD-related HCC is becoming also the sixth most common tumor globally and the second indication for liver transplantation in the United States (5). Intriguingly, MASLD-HCC may even occur in the absence of cirrhosis, further delaying its diagnosis (6, 7).

Nutritional and lifestyle habits as well as the metabolic status of patients may participate in determining the risk of HCC and modifying its prognosis (8). However, the mechanisms underlying this association are not completely elucidated. Thus, the present mini-review aims to deepen the role of metabolic comorbidities in predisposing to liver cancer in MASLD patients, emphasizing the correlation between HCC and the presence of cardiovascular abnormalities.

## 2 Common genetic signature between MASLD-HCC and lipid disorders

Ever increasing evidence testifies that the evolution of MASH towards HCC, with or without cirrhosis, is prompted by a plethora of common and rare inherited variations (9). Genetic variants which predispose to HCC are those in genes related to hepatic lipid handling including the *Patatin-like phospholipase domain containing 3* (*PNPLA3)*, the *Transmembrane 6 superfamily member 2* (*TM6SF2*) and *Membrane bound-o-acyltransferase domain-containing 7* (*MBOAT7)*, widely reported to be responsible for fatty loading of the liver (10). These polymorphisms alone and more so when combined in polygenic risk scores (PRSs), have been extensively associated with glucose reprogramming, tumorigenesis and metabolic switching towards aerobic glycolysis, mitochondrial failure and oxidative stress due to hepatic lipid overload (10, 11). In details, the prevalence of the three at-risk variants was 2.5-fold enriched in patients in the MASLD-HCC compared to non-carriers and their cumulative presence was associated with enhanced MASLD-HCC risk (11). Moreover, by applying a mendelian randomization approach, Dongiovanni and colleagues firstly reported that the co-presence of *PNPLA3*, *TM6SF2*, *MBOAT7*, and *GCKR* at-risk alleles, aggregated into a PRS, causally determines an increased susceptibility to develop severe chronic liver diseases, which reached about 13.4-fold for MASLD-HCC risk, as a consequence of their ability to induce hepatic fat accumulation (12). Accordingly, it has been demonstrated that the impact of each genetic variant on the odd to develop HCC is directly proportional to their ability to predispose to fatty liver (12, 13). For instance, the genetic variation encoding the p.I148M substitution in PNPLA3 which is the major inherited determinant of hepatic fat accumulation, is enabled to predispose to an until 5-fold higher risk to develop HCC even independently of fibrosis (14). Likewise, the common rs641738 close to *MBOAT7* has been pointed out as a modifier of the susceptibility to develop HCC even in the absence of cirrhosis as a consequence of its ability to induce giant lipid droplets (LDs) into the hepatocytes and other rare loss-of-function variants in the same gene have been found enriched in patients with MASLD-HCC (15). Therefore, a close relationship between circulating/hepatic lipids and hepatocarcinogenesis exists and genetic variants influencing them may be helpful in discriminating those MASLD patients at increased risk of HCC.

In keeping with this notion, other variants implicated in the remodeling of lipids and their release favor the switching from fatty liver towards cancer. Indeed, similarly to what occur in patients carrying the p.E167K allele of TM6SF2, even rare mutations with large effect size in the Apolipoprotein B (*APOB*) gene, have been correlated with hypobetalipoproteinemia and a protection against cardiovascular complications, but with more severe liver injuries (15). Indeed, ApoB participates to the VLDL gathering and modulates their secretion and loss-of-function variants blunt circulating lipoprotein concentrations. This event triggers severe fat depots’ formation due to VLDL retention and progressive MASLD up to HCC (16). As a consequence, a significant enrichment in pathogenic and truncating mutations in *APOB* gene has been identified in MASLD-HCC patients (15). In line with a possible causative role of lipid retention in hepatocytes in fostering HCC onset, even somatic mutations in *APOB* frequently occur during hepatocarcinogenesis (17). Hence, *APOB* alterations have been recently outlined as a prognostic biomarker for HCC (18).

Similarly, other variants influencing fasting lipids have been reported to increase the susceptibility to severe liver injuries. In particular, Dongiovanni and colleagues demonstrated that the proprotein convertase subtilisin/kexin type 7 (*PCSK7*) rs236918 G > C gain-of-function mutation coupled atherogenic dyslipidemia and acute coronary syndrome with MASH-fibrosis in a large histologically-characterized cohort of MASLD patients (19). Accordingly, it has been recently reported that this variant is enabled to modulate ApoB protein levels reinforcing the hypothesis of a possible role for PCSK7 in modulating lipid metabolism, LDs formation and liver damage (20). Likewise, genetically-determined aberrant expressions of PCSK9, another member of the proprotein convertase subtilisin/kexin family, have been correlated with hereditary hypercholesterolemia, severe steatosis and cardiovascular abnormalities (21–23). However, the impact on hepatocarcinogenesis of inherited variants in these two genes is still unexplored.

Moreover, the rs599839 A>G variant localized in the 1p13.3 locus related to lipid traits reduced the risk of coronary artery disease (CAD) and dyslipidemia, favoring in turn the incidence of liver cancer, poor prognosis, reduced overall survival and advanced tumor stages in HCC patients. The likely mechanism behind this association is due to the overexpression of the oncogene Proline And Serine Rich Coiled-Coil 1 (*PSRC1*), and of Sortillin 1 (*SORT1*), both involved in distinct pathways. The former regulates cell proliferation participating to microtubule destabilization and spindle assembly, while the latter modulates circulating lipid profiles, reinforcing lipoprotein clearance, turnover and dismissal. Hence, similarly to the aforementioned mutations, it disentangles the risk of atherogenic dyslipidemia and hepatocarcinogenesis, whereby enhancing HCC incidence, but ameliorating serum lipid profile (24). Accordingly, hepatic PSCR1 expression associated with increased HCC recurrence, and its overexpression was observed in hepatoma tissues compared to the adjacent ones and correlated with reduced survival (25, 26). Conversely, the Neurotensin (NTS) rs1800832 variant has been described to enhance the susceptibility to coronary heart diseases, and predispose to advanced fibrosis and HCC in MASLD patients. This polymorphism modulates circulating pro-NTS, a peptide mainly released from entero-endocrine N cells of the gastrointestinal tract which facilitates intestinal fatty acid absorption and its effects are mediated by three neurotensin receptors (NTSRs), one of which is SORT1. Exaggerated NTS expression has been described in human HCC samples, where it mediates hepatocyte proliferation and epithelial mesenchymal transition (EMT), being tumors more aggressive and promoting tumor invasion, spreading and metastasis (27). Even more, elevated pro-NTS circulating levels were significantly correlated with the presence of HCC (28). Therefore, we could hypothesize that other inherited mutations exerting large effects on lipoprotein metabolism may be useful to classify patients with MASLD according to their relative risk of developing cardiovascular *vs*. liver-related events.

Other genetic risk factors may play a crucial role in the transition from simple steatosis to MASH and cancer, whereby modulating the lipid composition and the size of the LDs. For instance, the rs35568725 (p.S251P) variant in Perilipin-2 (*PLIN2*) gene, which regulates the stability and the remodeling of LDs and VLDL lipidation, predisposes to severe insulin resistance (IR) and atherosclerosis. This mutation conveys the risk of severe hepatic disease favoring the accumulation of small LDs in hepatocytes (29, 30). The pro-carcinogenic role of PLIN2 is still under investigation. However, it has been found up-regulated in HCC samples, gaining prognostic value and influencing adverse outcomes (31). Likewise, also PLIN5, another member of the perilipin family, is highly expressed in livers isolated from HCC patients and diethylnitrosamine (DEN)-treated mice (32). The rs884164 in *PLIN5* downregulates its protein expression, blunting the number of contacts between mitochondria and LDs and lipid degradation. This variant is associated with a poorer outcome following myocardial ischemia, and enhanced oxidative stress in cardiomyocytes (33, 34). Alongside, other inherited defects in autophagic processes, mainly in lipophagy, accelerates LD accumulation (35). Although the impact of the variants in *PLIN2* and *PLIN5* genes on liver cancer is still under definition, we cannot rule out that they may define disease progression to cancer and therapeutic response, due to their contribution to LD number, size and composition. Indeed, it has been hypothesized that LDs sequester anti-cancer drugs, hampering their efficacy. Moreover, since augmented LD biogenesis have been reported in different neoplastic conditions, this makes LDs novel suitable targets for anticancer drugs and for the development of new dyes for cancer cells imaging (36, 37).

## 3 Role of hyperlipidemia in HCC development and evolution

### 3.1 Fatty acids and lipid droplets metabolism in the modulation of HCC risk

MASLD is a complex disorder, whose pathogenesis involves multi-parallel hits, either environmental or genetic factors. TGs, diglycerides, and ceramides overload trigger endoplasmic reticulum (ER) stress and mitochondrial dysfunction, causing both calcium (Ca2+) and reactive oxygen species (ROS) enrichment, which are directly involved in DNA mutagenesis, activation of oncogenes or inhibition of onco-suppressors (11, 38, 39).

Several studies revealed that hepatic LDs accumulation and *de novo* lipogenesis (DNL) play a crucial role in HCC development. Notably, the inhibition of key enzymes involved in lipogenic pathways, such as stearoyl-CoA desaturase (SCD), fatty acid synthase (FASn), and acetyl-CoA carboxylase (ACC), has been linked to a reduced presence and proliferation of cancer stem cells (CSCs), which are critical for tumor growth and progression, in both *in vitro* and *in vivo* models (40, 41). Moreover, free fatty acids (FFAs) derived from adipose tissue lipolysis, combined with those synthesized through DNL, may accelerate hepatocytes’ degeneration, and promote tumor escape mechanisms by activating anti-apoptotic programs. Palmitic acid (PA) treatment affects insulin signaling, enhances β-oxidation, exacerbates ROS content, and turns on c-Jun NH2-terminal kinase (JNK), a constitutively activated factor in HCC. The accumulation of oleic acid (OA) and PA promotes liver cancer by suppressing Pten, an inhibitor of Pi3k/Akt/mammalian target of rapamycin (mTOR) signaling (42).

Notwithstanding, it has been observed that HCC has a wide molecular and phenotypic variability. Indeed, T2D, obesity, or MASLD may induce the development of “oxidative” HCCs, in which FFAs are catabolize through β-oxidation and increase ATP availability. This mechanism may favor cell growth and mainly involve the activation of PPARα, the master regulator of FFA degradation, and of WNT/β-catenin oncogenic cascade (43). In keeping with these findings, Tian et al. has demonstrated that LD breakdown (lipophagy) was unexpectedly involved in carcinogenesis in hepatoma cell lines, MASLD murine models, and MASLD-HCC human samples (44).

Even more, LD formation and their dimension (micro/macro-LDs) hover a crucial role in HCC metastasis, stemness and response to therapy. Interestingly, besides stocking energy sources, LDs work as a buffering system incorporating lipotoxic species. It has been reported that long-term Sorafenib exposure, the first-line treatment for advanced HCC, impairs β-oxidation, causing the accumulation of FAs. In turn, toxic FAs are incorporated into LDs, favoring their biogenesis and enlargement, and increasing the HCC susceptibility to became drug resistant. This adverse effect has been recently attributed to the upregulation of AKR1C3 protein, which promotes LD shaping and accumulation, insofar as the selective AKR1C3 inhibition improved Sorafenib resistance in HCC cell lines (45).

PLIN1/2/3 proteins mediate LDs–mitochondria crosstalk, regulating LDs’ expansion and disposal. Notably, PLIN1, PLIN2 and PLIN3 are differentially expressed during tumorigenesis and usually dwell on the LDs’ surface according to the LDs’ dimension. PLIN2 and PLIN3 mainly coat small LDs and are commonly overexpressed at early stages of HCC, as the LD dimension allows rapid dynamics between synthesis and consumption to sustain phases of cell proliferation or metabolism. PLIN1 expression is lost during hepatocarcinogenesis and may reflect the differentiation grade of hepatocytes (46).

The activation of SREBP1 via PI3K/Akt/mTOR further contributes to neoplastic steatogenesis and PLINs expression (47). These studies pointed out a differential role of micro/macro-LDs in HCC onset and progression, although details on the mechanisms and respective roles need to be addressed in the future.

### 3.2 Cholesterol and HCC

Recent clinical and preclinical evidence supported the notion that an excessive cholesterol intake may represent an independent risk factor for liver cancer development in the context of MASLD, even irrespectively of cirrhosis (48–50). However, a precise definition of the likely mechanism behind this association remains to be elucidated. In different rodent models of obesity and diabetes, it has been demonstrated that hyperinsulinemia stimulates induction of new cholesterol synthesis, through sterol regulatory element-binding protein 2 (SREBP2) activation, low-density lipoprotein receptor (LDLR) up-regulation, and the shutdown of its conversion into bile acids, thus leading to the hepatic accumulation of free cholesterol. Cholesterol overload, in turn, favors lipotoxicity into the hepatocytes, mediating the progression to MASH (48). In details, cholesterol over-storage into the organelles as ER and mitochondria generates aberrancies in the intracellular membranes, ROS production, ATP depletion and hepatocyte degeneration. Overall, these events predispose to a more prone pro-inflammatory milieu, priming the transition from uncomplicated steatosis to steatohepatitis and fibrosis (48, 51, 52). Indeed, cholesterol uptake mediated by the scavenger receptor (SR-A) or by CD36 and by lectin-like oxidized LDL receptor-1 (LOX-1), exerts a dual role on Kupffer cells and hepatic stellate cells (HSCs), respectively, thus promoting their activation, the release of pro-inflammatory cytokines, oxidized mtDNA and pro-cancerous factors (53). Accordingly, DEN administration to mice fed a high fat high cholesterol (HFHC) diet induced more severe oxidative DNA damage, non-synonymous somatic mutations, numerous and large liver tumors compared to littermates treated with DEN alone (54). In addition, rodents fed HFHC displayed a pronounced gut microflora dysbiosis and fecal microbiota transplantation in germ-free mice favored the onset of steatosis, inflammation, oxidative stress and cell proliferation. On the contrary, microbiota manipulation to restore the eubiosis as well as cholesterol lowering agents as atorvastatin might be effective for HCC prevention (55).

It has been largely reported that free cholesterol depots are accumulated in patients with MASH, due to the imbalance between its biosynthesis, conversion and excretion and free cholesterol levels correlate with the severity of the disease (56, 57). High cholesterol assumption has been proven to enhance the susceptibility to HCC in a population-based study among 14,407 participants conducted in the United States, but serum cholesterol concentrations were not associated with a higher risk of cirrhosis or liver cancer (49). Conversely, Carr and colleagues found a positive correlation between plasma cholesterol, lipoprotein levels and tumor growth, cell invasion and aggressiveness (58). Likewise, a nested case-control study within the Scottish Primary Care Clinical Informatics Unit (PCCIU) database demonstrated that statins assumption had a protective effect on HCC risk (hazard ratio HR, 0.48; 95% CI, 0.24–0.94) (59). In addition, statin users had 15% lower hazard ratio (HR) to develop a new liver disease, 28% lower HR of liver-related death and a 42% lower HR of HCC, showing a significant hepatoprotection in time and dose dependent manner (60). Similar benefits of statin use have been reported even in patients with chronic hepatitis B (CHB) (61).

Intriguingly, Ma and collaborators demonstrated that cholesterol levels may regulate also tumor immune microenvironment, whereby affecting natural killer and CD8+ T cells (62). In particular, these authors demonstrated that cholesterol-enriched tumors progressively upregulate the programmed death receptor 1 (PD-1), thus inducing CD8+ cell death and the loss of cell growth control. On the contrary, cholesterol-lowering agents, as simvastatin, prime the anti-tumor immunity mimicking the effect of the novel developed PD-1/Programmed death ligand 1 (PD-L1) inhibitors (63).

All-in-all, to further dissect the impact of cholesterol on the incidence of HCC and shed a light on the possible use of lipid biomarkers as a predictive sign of cancer, more investigations will be warranted.

## 4 Prognostic value of metabolic markers in MASLD-HCC patients

Emerging evidence outlines that metabolic factors, including dyslipidemia, may increase the susceptibility to develop HCC, especially in population with low prevalence of viral hepatitis (64). Indeed, the peculiar pro-inflammatory and lipotoxic milieu that characterizes the hepatic tissue of MASLD patients, influences the risk of HCC and adverse prognosis. However, the magnitude and the direction of this association is still under definition. In addition, except for the total cholesterol, only few studies reported an association between circulating LDL, HDL and TGs and hepatocarcinogenesis (65, 66). In this context, the idea to possibly exploit lipid biomarkers as an edge of the disease progression, became attractive.

For instance, neutrophil-to-HDL-C ratio (NHR) parameter has been recently proposed as prognostic indicator of mortality in HCC patients, exceeding the power of each single marker alone (67). Indeed, HDL cholesterol may play discrete anti-inflammatory and anti-oxidant functions and its reduction has been correlated with the presence of different metabolic disorders (68, 69), including HCC (70). Conversely, neutrophils constitute a precocious innate immune response to injured tissues, being recruited directly in proximity to the damage and parallelly increasing their count (71). Therefore, the evaluation of this composite marker may be representative of a severe disease status, overall reflecting both inflammation and lipid metabolism. Accordingly, in the last years, various applications for the NHR have been screened, as an index of acute ischemic stroke (AIS) (72), or as a predictor of clinical outcomes of all-causes of cardiovascular mortality (73). In addition, low HDL cholesterol has been recently reported as novel marker to predict HCC in 1,234 MASLD patients by Crudele and collaborators (74). However, further population-based studies are required to better define its sensitivity and sensibility in foreseeing MASLD-HCC.

Given the strong impairment in hepatic parenchyma, an abnormal pattern of serum lipoproteins as ApoA1 and ApoA2, has been observed in patients with HCC (75, 76). For this reason, it has been pointed out ApoA1 as a candidate biomarker for early diagnosis, prognosis, and monitoring of HCC, in patients affected by viral hepatitis C (77). However, one of the main issues is that ApoA1 is not a sensitive or specific biomarker enough to separate HCC from chronic liver diseases, in which hepatic dysfunction occurs. Nonetheless, these findings further corroborate the association between a dyslipidemic profile and the presence and/or the risk of liver cancer (78, 79). Accordingly, we recently demonstrated that the low serum lipoprotein (Lp(a)) concentrations correlated with transaminase elevation and increased risk of developing cirrhosis in MASLD patients, thus representing a novel lipid biomarker to reliably predict severe liver disease (80). Alongside, Lp(a) has been proposed also as an indicator of tumor recurrence after curative resections since it represents an hallmark of the residual hepatic function (81).

Concerning other lipid-related biomarkers, preclinical and *in vitro* studies demonstrate that serum and hepatic PCSK9 levels induced by glucose exposure, alter the response of hepatoma cells to cancer therapy (82). In addition, the high availability of glucose in tumor tissues fosters PCSK9 elevated secretion, which favors, in turn, hypercholesterolemia (82).

Considering the metabolic reprogramming that characterizes hepatoma cells, it is conceivable that lipidomic-based studies may provide novel biomarkers. For instance, a downregulation of plasma phosphatidylcholine species (83) as well as of ceramides (84) has been reported to have an acceptable predictive performance for HCC, whereas the enhancement of Sphingosine 1-phosphate (S1P), the prevalent hepatic sphingolipid, supports tumor growth (85). Likewise, the assessment of the typically aberrant fatty acid metabolism (FAM), which supports cancer cell proliferation, may be useful to construct prognostic risk models for HCC (86).

Alongside novel lipidomics approaches, also recent metabolomics studies on blood samples from subjects with HCC revealed aberrant circulating profiles of glucose, lactic acid, retinol, bile acids and amino acids (including alanine, glutamine, 1-methylhistidine, lysine, and valine), mainly as a consequence of the unique HCC metabolism (87). Notably, a deep understanding of the utility and reliability of these omics biomarkers is necessary to consider them for their introduction into the clinical practice. Even more, further validation analyses in larger cohorts of patients are required to understand whether these serum metabolites may be helpful in diagnosing HCC and in stratifying MASLD-HCC patients according to their prognosis. In this context, an integrated view of the metabo-lipid signature of HCC patients is now emerging as a diagnostic opportunity for Alpha-fetoprotein (AFP) false-negative subjects (88).

## 5 Cardiovascular risk in patients affected by MASLD-HCC

### 5.1 Cardiovascular complications, dyslipidemia and type 2 diabetes in MASLD-HCC patients

As extensively reported, the amount and the diameter of circulating LDL cholesterol is one of the primary risk factors that predispose to vascular atherosclerotic diseases in both men and women (89). In details, numerous, small, dense and oxidized LDL particles constitute the more noxious elements in the etiology of CAD. Nevertheless, the causal relationship between HCC and cardiometabolic risk factors is still under investigation. Indeed, conflicting results are available regarding the association between LDL levels and the odd of liver cancer. On one hand, it has been demonstrated that LDL cholesterol loading into intracellular organelles prompts oxidative and ER stress, thus creating a more pro-oncogenic microenvironment (90), on the other, some epidemiological studies described a significant association between relatively low levels of LDL cholesterol (<100 mg/dL) and the risk of incident cancer (91, 92). In keeping with the latter observation, a reduction of the overall mortality in HCC patients with hypertriglyceridemia (HR, 0.38; 95% CI, 0.26 to 0.55) and hypercholesterolemia (HR, 0.50; 95% CI, 0.37 to 0.67) has been reported by Chiang et al. (66).

Notwithstanding, Bertero and colleagues determined that patients affected by cardiovascular diseases (CVD) are more likely prone to develop malignancies, sustaining a possible shared biology (i.e., inherited or acquired predisposing factors, inflammation, stress and angiogenesis) that primes tumors and CVD development (93). Hence a common multi-factorial pathogenic substrate, involving T2D, dyslipidemia, hypertension, and obesity for both diseases has been postulated (94). Accordingly, Banke and collaborators further corroborate this notion, describing that patients with chronic heart failure have an increased susceptibility to develop any-type of cancers, with an incidence ratio of 1.24 (95). The other side of the coin is that fatal cardiovascular complications and heart involvement in liver cancer is considered a poor prognostic indicator, reducing the survival and cardiac metastasis occurring in 10% of HCC diagnosis (96). Therefore, the overall clinical framework of HCC patients, the use of cholesterol-lowering medications, other therapeutic and dietary indications, and the inference of genetic variations should be rigorously taken into account to evaluate these associations.

A greater unified opinion outlines the causal relationship between poor glycemic control/T2D and the incidence of HCC, thus pointing out the need of achieving the goal of a better glucose management to reduce long-term complications of diabetes in these patients (66, 91, 97). Several epidemiological studies supported this evidence (98–102) and a meta-analysis across 26 studies claimed an almost doubled risk of HCC in patients with T2D of different ethnicity and geographic localization (103).

In particular, T2D represents an independent risk factor for HCC, in both men and women and this association became stronger with prolonged T2D duration and more so in the co-presence of other comorbid metabolic conditions (97). Indeed, hyperglycemia, raised levels of insulin and insulin-like growth factor 1 (IGF1) display proliferative and oncogenic effects and they worsen the already existing cardiometabolic risk factors exasperating chronic inflammation, endothelial dysfunction, oxidative stress and DNA damage thus creating a favorable tumor microenvironment. In this context, intestinal dysbiosis and microbial metabolites may promote the raising of pro-inflammatory, pro-coagulative and pro-fibrotic circulating molecules, as the endotoxins (104, 105). Notably, it has been also reported that T2D and HCC share almost 336 differentially methylated genes (DMGs), including 86 co-methylated DMGs, mainly involved in glycosaminoglycan biosynthesis, fatty acid and metabolic pathways, whereas 250 DMGs with a different methylation direction, enriched in the Sphingolipid metabolism and immune signaling, thus corroborating a common pathophysiology between these two diseases (106). Otherwise, endothelial dysfunction *per se* is responsible for vasoconstriction and platelet aggregation fueling pro-inflammatory mediators release, hypertension, diabetic nephropathy and cardiovascular diseases, fostering a ‘vicious cycle’ (107). Hence, T2D appears as the common denominator between hepatocarcinogenesis and cardiovascular abnormalities, by predisposing to both advanced lesions, poor outcomes, vascular complications and higher mortality rate (108). Nonetheless, irrespectively of coexisting factors, IR is the major determinant of severe hepatic fibrosis, which is an excellent prognostic indicator of HCC onset (109). Alongside hyperinsulinemia has been reported to be responsible for the induction of more aggressive cancer phenotypes and poor prognosis even in other types of tumors (110).

Finally, the increasing number of metabolic comorbidities, including dyslipidemia, T2D, obesity and hypertension has been associated with enhanced predisposition to HCC, reaching an 8.1-fold increased HCC risk (95% CI, 2.48–26.7) in the co-presence of these four cofactors (97).

### 5.2 Other cardiometabolic risk factors influencing the risk of MASLD-HCC: the role of abdominal obesity and hypertension in modifying hepatocarcinogenesis

As abovementioned, it is now widely acknowledged that obesity, hypertension, glucose intolerance and T2D frequently co-occur with hepatic steatosis, contribute to its progression and have been associated with both cardiovascular and HCC risk in MASLD patients (111).

Obesity has been increasingly identified as a major driver in the evolution of MASLD up to HCC. Substantial adipose tissue deposits introduce significant complications for the diagnosis and screening of HCC with both invasive and non-invasive techniques. The presence of subcutaneous and visceral fat can hinder the detection of hepatic lesions by ultrasound, the standard protocol for HCC surveillance, as well as it can make challenging the interpretation of images obtained with higher resolution equipment, such as MRI and CT. Additionally, in liver biopsies, the accessibility to the liver can be limited by abdominal fat accumulation, raising the risk of procedural-related complications (112).

Two large population studies carried out in Denmark and US, including 43,965 and 900,000 cases, respectively, have shown that liver cancer development was by around 2-fold higher in obese patients compared to the general population. Furthermore, the relative risk of dying from liver cancer increased by 1.68 times for women and 4.52 times for men with a BMI>30 kg/m^2^ compared to a reference group with a median BMI>21.8 kg/m^2^ (113, 114). Recently, Rustgi and collaborators conducted a retrospective study in a large cohort of 98,090 newly diagnosed MASLD patients eligible for bariatric surgery, spanning from 2007 to 2017. The authors found that MASLD subjects who underwent bariatric interventions exhibited a lower rate of obesity-related cancer, including HCC, compared to those who did not undergo the surgery (115). These findings were partially corroborated by Saito et al. who tested the efficacy of a multidisciplinary weight loss program (WLP), including the nutritional assessment and physical exercise, in HCC patients with a high BMI (≥25 kg/m^2^) before they underwent hepatic resection. Despite WLP did not impact on HCC recurrence or progression, it improved the immune status and liver function (116), thereby supporting that interventions like bariatric surgery and weight loss may potentially aid to mitigate HCC risk and to ameliorate its management.

Hypertension represents the most prevalent cardiometabolic risk abnormality in MASLD patients, diagnosed in up to 50% of cases. The use of antihypertensive drugs, which include angiotensin-converting enzyme inhibitors (ACEi), calcium channel blockers (CCBs), beta-adrenoceptor blockers (BBs), angiotensin receptor blockers (ARBs) and thiazide diuretics, is a critical component in MASLD management, since hypertension showed an independent association with HCC risk and poor prognosis (117). Nonetheless, it is still unclear whether and how antihypertensive drugs may modify the risk of HCC or may limit the efficacy of anti-cancer therapies (118, 119). For instance, captopril, an ACEi, reduced hepatic fibrosis and prevented progression towards HCC development in murine models of liver cancer (119). Conversely, Zhang and coworkers reported that ACE inhibitors may compromise the effects of anti-angiogenic drugs in HCC mouse models (120), thus supporting the necessity for a rigorous monitoring and control of hypertension in MASLD-HCC patients to optimize treatment efficacy and outcomes.

A large meta-analysis collecting data from 1976 to 2017 in 12 countries (a total of 526,336 participants) have shown that hypertension awareness, treatment, and control have been improved in high-income states (121). However, in the past decades, an alarming data has emerged concerning the ever-increasing percentage of MASLD patients who suffered of uncontrolled hypertension, which *per se* represents a negative predictor of CVD-related mortality (121, 122). Multiple factors have been recognized as major responsible behind this trend, including non-adherence to treatment guidelines, physician inertia, medication non-compliance, or limited health literacy (122), underlining the urgent need to integrate comprehensive hypertension screening and management strategies into the care protocols for MASLD patients.

## 6 Our novel findings regarding the prevalence of carotid plaques in HCC patients

In the attempt to provide further clinical evidence for the surveillance of HCC patients, we explored the relationship between the risk of hepatocarcinoma and cardiometabolic comorbidities in a large cohort of biopsied-proved MASLD patients (n=1538; Overall cohort). The Overall cohort was enrolled at the Fondazione IRCCS Cà Granda Ospedale Maggiore Policlinico of Milan, as previously described (11, 24).

The Overall cohort (n=1538) included n=1452 MASLD subjects with different stages of the disease (Hepatology service cohort) and n=86 MASLD-HCC individuals (**Table 1**). Histological evaluation was staged according to NAFLD activity score (NAS) by Kleiner et al. for steatosis, necroinflammation and ballooning degree. MASH was diagnosed when (a) steatosis, (b) lobular inflammation and (c) ballooning were concomitantly present. Fibrosis stage was defined according to the recommendations of the NAFLD Clinical Research Network (123). Informed written consent was obtained from each patient and the study protocol was approved by the Ethical Committees of the Fondazione IRCCS Ca’ Granda, Milan and it was in conformity with the ethical guidelines of the 1975 Declaration of Helsinki.

**Table 1:** Demographic, anthropometric and clinical features of the Overall cohort (n=1538), stratified according to class enrollment criteria (n=1452, Hepatology service cohort and n=86, MASLD-HCC).

	Overall cohort (n=1538)	Hepatology service (n=1452)	MASLD-HCC (n=86)	*P* value*
Sex, M	808 (52.5)	742 (51)	66 (77)	**<0.0001**
Age, years	49 ± 0.34	48 ± 0.33	67 ± 1.42	**<0.0001**
BMI, kg/m2	34.5 ± 0.23	34.7 ± 0.23	28.8 ± 1.12	**<0.0001**
Obesity, yes	925 (60.1)	904 (62.3)	21 (24.4)	**<0.0001**
IFG/T2D, yes	402 (26)	351 (24.1)	51 (60)	**<0.0001**
Glucose, mg/dL	102.9 ± 0.8	101.6 ± 0.8	131.5 ± 3.8	**<0.0001**
Insulin, IU/ml	20.7 ± 0.8	20.6 ± 0.8	24.3 ± 5.9	0.35
HOMA-IR	5.4 ± 0.26	5.2 ± 0.25	11.04 ± 1.9	**<0.0001**
Total cholesterol, mmol/L	197.5 ± 1.14	198.8 ± 1.15	164.9 ± 5.7	**<0.0001**
LDL cholesterol, mmol/L	123.3 ± 1.03	124.3 ± 1.05	99.2 ± 5.2	**<0.0001**
HDL cholesterol, mmol/L	49.9 ± 0.4	49.9 ± 0.4	49.9 ± 1.99	0.98
Triglycerides, mmol/L	142 ± 2.4	143.5 ± 2.4	107.2 ± 12.1	**0.003**
Lp(a), nmol/L	32.5 ± 1.6	32.9 ± 1.7	24.7 ± 6.8	0.22
Carotid IMT, mm	0.83 ± 0.009	0.83 ± 0.009	0.93 ± 0.04	**0.01**
Statin, yes	148 (9.6)	137 (9.4)	11 (13.4)	0.39
ALT, IU/l	34 {21–57}	33 {20–57}	43 {27–54}	0.29
AST, IU/l	26 {19–39}	25 {19–38}	47 {27–72}	**<0.0001**
GGT, IU/l	41 {22–82}	39 {22–78}	84 {49–176}	**<0.0001**
LDH, U/l	188 {221–263}	184 {109–254}	247 {179–335}	**0.008**

Values are reported as mean ± SE, number (%) or median {IQR}, as appropriate. BMI, body mass index; IFG, impaired fasting glucose; T2D, type 2 diabetes; IMT, intima-media thickness. Variables with skewed distribution were logarithmically transformed before analyses. IFG defined as fasting glucose >110 mg/dL. Mean carotid artery intima-media thickness (IMT), an index of the early atherosclerotic process, was determined by high−resolution B-mode ultrasonography with a 7.5‐MHz transducer. Values of IMT represent the mean IMT on the left and right sides. The presence of plaques was defined as a focal carotid thickening >1.2 mm. Systolic and diastolic blood pressures were measured twice on the same day, and the mean values were used for analysis. The presence of hypertension was defined when systolic blood pressure was over 140 mm Hg or diastolic blood pressure was over 90 mm Hg more than twice or in subjects treated with antihypertensive medication (80). *p<0.05 was considered statistically significant at two-way ANOVA (MASLD-HCC vs Hepatology service cohort). Bold values are those statistically significant.

MASLD-HCC patients, were predominantly older men and overweight, exhibited higher circulating transaminase (AST and GGT) and lactate dehydrogenase (LDH) levels compared to those belonging to the Hepatology service cohort (p<0.001, **Table 1**).

Moreover, a reduced total cholesterol, LDL cholesterol and TGs together with higher fasting glucose concentration and HOMA-IR values were observed in MASLD-HCC patients compared to the Hepatology service cohort (p<0.001, **Table 1**). These findings were consistent with those of Cao J et al, who found a strong association between low-LDL cholesterol levels and HCC occurrence in a mendelian randomization analysis across 212,453 individuals. Similarly, this correlation was confirmed by Li M. et al. in a multicentric, prospective study including 137,884 participants, who even reported that patients with a poor glycemic control showed a higher risk of liver cancer (91, 92).

**Figure 1:**
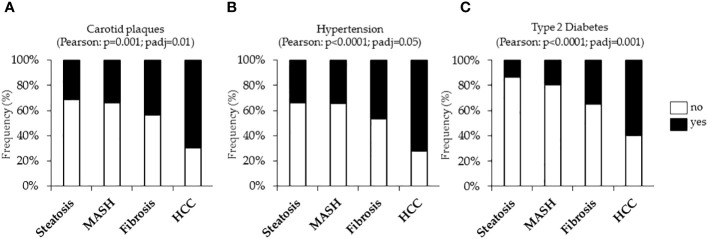
Cardiometabolic risk factors, including carotid plaques and hypertension and type 2 diabetes (T2D) were evaluated in the Overall cohort (n=1538) stratified according to the liver disease severity. Contingency analysis showed the frequency distribution of carotid plaques **(A)**, hypertension **(B)** and T2D **(C)** in the Overall cohort. p<0.05 was considered statistically significant. P values were further adjusted for age as confounding factor.

The incidence of cardiovascular complications was firstly analyzed in the Overall cohort, stratified according to histological liver damage. In the Overall cohort, n=389 cases (25.3%) had uncomplicated steatosis, n=280 (18.2%) presented MASH, n=783 patients (50.9%) had MASH plus fibrosis (referred to as fibrosis) and n=86 (5.6%) developed HCC. In keeping with the notable increase in carotid intima-media thickness (IMT) observed in MASLD-HCC patients compared to the Hepatology service cohort (p<0.05, **Table 1**), we showed that the frequency of carotid plaques was significantly higher MASLD-HCC subjects compared to those with a less severe disease (carotid plaques: 75% vs 30%, 33.9% and 43.5%; p=0.001 at Pearson correlation analysis; padj=0.01 after the adjustment for age **Figure 1A**), despite the lower circulating lipids detected in these patients (**Table 1**). Alongside this, a marked increase in the incidence of hypertension and T2D were found in MASLD-HCC individuals, and their frequency progressively increased with MASLD worsening. Specifically, hypertension was present in 76.5% of MASLD-HCC patients compared to 33.6%, 34.2%, and 46.8% in earlier stages of the disease (p<0.0001 at Pearson correlation analysis; padj=0.05 after the adjustment for age **Figure 1B**), while T2D reached the 58.8% in HCC cases *vs.* 13.6% in uncomplicated steatosis, 19.15% in MASH and 34.8% in fibrosis (p<0.0001 at Pearson correlation analysis; padj=0.001 after the adjustment for age **Figure 1C**), thereby suggesting that these comorbidities may track the course of MASLD, exerting a significant impact on carotid plaque formation.

**Figure 2:**
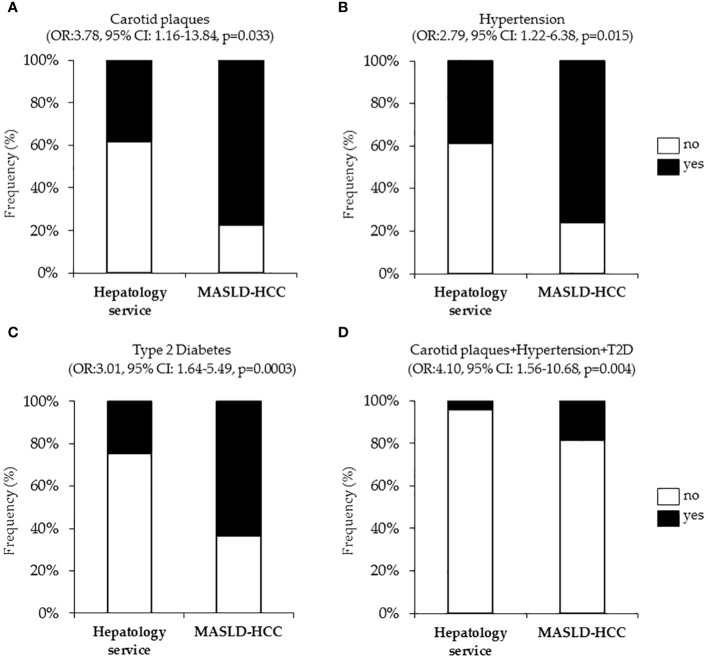
Association of cardiometabolic risk factors (carotid plaques and hypertension, T2D), with MASLD-HCC risk in the Overall cohort (n=1538). Nominal logistic regression models adjusted for confounding factors correlating MASLD-HCC occurrence with carotid plaques **(A)**, hypertension **(B)**, T2D **(C)** and cumulative presence of plaques, hypertension and T2D **(D)**. OR: odds ratio, p<0.05 was considered statistically significant.

It is well established that T2D, hypertension and cardiovascular diseases are closely interconnected together to the point that their coexistence in the same individual exacerbates endothelial dysfunction, vascular inflammation and fibrosis, arterial remodeling, and atherosclerosis (124). However, less is known regarding their link with the occurrence of liver cancer. Intriguingly, at nominal logistic regression analysis, adjusted for age, sex, BMI, T2D, and statin use, we revealed a strong correlation between the presence of carotid artery plaques and MASLD-HCC (OR:3.78, 95% CI: 1.16–13.84, p=0.033, **Figure 2A**). Additionally, hypertension (OR:2.79, 95% CI: 1.22–6.38, p=0.015, **Figure 2B**) resulted independently associated with MASLD-HCC risk at multivariate analysis after the adjustment for age, sex, BMI, T2D and active smoking. Again, T2D associated with more than six-fold increase of MASLD-HCC risk at nominal logistic regression analysis adjusted for age, sex and BMI (OR:3.01, 95% CI: 1.64–5.49, p=0.0003, **Figure 2C**), thus aligning with and strengthening previously reported studies (105, 125). Moreover, we further revealed that the combined prevalence of carotid plaques, hypertension, and T2D was substantially higher in MASLD-HCC patients (18.2% in MASLD-HCC *vs*. 4.2% in the Hepatology service cohort, **Figure 2D**). Therefore, in our cohort, the risk of liver cancer was significantly amplified by the co-occurrence of these comorbidities (OR:4.10, 95% CI: 1.56–10.68, p=0.004), compared to their presence alone.

In sum, our data identifies a paradox in MASLD-HCC patients who exhibited lower circulating lipid levels and higher incidence of plaque formation compared to individuals at different MASLD histological stages. This discrepancy could stem from various factors. Firstly, among MASLD-HCC patients, those developing plaques exhibited higher serum total cholesterol (169 mmol/L *vs* 148 mmol/L), LDL (163 mmol/L *vs* 78 mmol/L), and TGs (117 mmol/L *vs* 99 mmol/L) concentrations compared to subjects with MASLD-HCC without plaques. This observation possibly suggests that MASLD-HCC patients developing plaques have a worsened lipid profile which may contribute to their heightened cardiovascular risk. Additionally, at the time of HCC diagnosis, many patients may be in a deteriorated state of health, often with significant weight loss, which inherently affects lipid levels. Moreover, the advanced liver dysfunction, characteristic of HCC, impacts the liver’s capacity to produce lipoproteins and TGs, leading to altered circulating lipid profiles. Equally important is the prevalence of cardiometabolic conditions such as T2D, hypertension, insulin-resistance, and hyperglycemia in our MASLD-HCC cohort, which may predominantly contribute to plaque formation, possibly overshadowing the influence of lipids. Finally, we cannot rule out that genetic predispositions and the usage of cholesterol-lowering medications are likely to have significant effects on both the lipid metabolism and cardiovascular risk profiles. Collectively, our findings may contribute to the growing body of evidence linking cardiometabolic factors with HCC and highlight the necessity for comprehensive clinical evaluations in patients with MASLD.

## 7 Conclusion

Given the ever-increasing global incidence of HCC, effective strategies of prevention, surveillance and personalized therapeutic approaches are still mandatory. In particular, since around 40% of HCC cases are attributable to metabolic disorders, including MetS, T2D, obesity, and hyperlipidemia (126, 127), preventive approaches aimed to counteract the development of cardiometabolic risk factors may be useful in limiting the oncologic predisposing background. In patients with MASLD-driven HCC, we could postulate that the introduction of an integrated dietary regimen and more healthy lifestyles, including physical exercise may be useful to reduce the incidence of advanced stages of disease and HCC. However, conflicting results are currently available and further studies are required to better dissect the association between metabolic risk factors, cardiovascular diseases and HCC onset. To shed light on this steamy landscape, we carry out an observational study in our large biopsied MASLD cohort, including 86 MASLD-HCC. Our findings sustained the critical role of T2D and hypertension in enhancing HCC risk and emphasized the pivotal contribution of cardiometabolic comorbidities, particularly when they coexist, on disease prognosis. Interestingly, while our data revealed that MASLD-HCC patients showed lower circulating lipid levels, a paradoxical increase in IMT thickness and plaque formation was observed. Nonetheless, within the MASLD-HCC subgroup, patients with plaques present a worse lipid profile compared to HCC subjects without plaques, potentially elevating their cardiovascular risk. Indeed, some population-based studies have supported our statements, pointing out that patients affected by HCC are more vulnerable to develop stroke/cardiovascular events than patients with other types of cancers (128–130). Therefore, these findings highlighted the complexity of lipid profiles in MASLD-HCC and the importance of a detailed assessment of cardiovascular risk, advocating for an integrated approach which considers the spectrum of both metabolic and cardiovascular factors.

## Author contributions

MM: Conceptualization, Investigation, Resources, Writing – original draft, Writing – review & editing. ML: Conceptualization, Investigation, Writing – original draft. PD: Conceptualization, Funding acquisition, Investigation, Resources, Supervision, Writing – original draft, Writing – review & editing.
